# Expression of Hemolysin Is Regulated Under the Collective Actions of HapR, Fur, and HlyU in *Vibrio cholerae* El Tor Serogroup O1

**DOI:** 10.3389/fmicb.2018.01310

**Published:** 2018-06-19

**Authors:** He Gao, Jialiang Xu, Xin Lu, Jie Li, Jing Lou, Hongqun Zhao, Baowei Diao, Qiannan Shi, Yiquan Zhang, Biao Kan

**Affiliations:** ^1^State Key Laboratory of Infectious Disease Prevention and Control, National Institute for Communicable Disease Control and Prevention, Chinese Center for Disease Control and Prevention, Beijing, China; ^2^Key Laboratory of Cleaner Production and Integrated Resource Utilization of China National Light Industry, School of Food and Chemical Engineering, Beijing Technology and Business University, Beijing, China; ^3^School of Medicine, Jiangsu University, Zhenjiang, China

**Keywords:** *Vibrio cholerae*, regulation, HlyA, quorum sensing, HapR, Fur, HlyU

## Abstract

The biotype El Tor of serogroup O1 and most of the non-O1/non-O139 strains of *Vibrio cholerae* can produce an extracellular pore-forming toxin known as cholera hemolysin (HlyA). Expression of HlyA has been previously reported to be regulated by the quorum sensing (QS) and the regulatory proteins HlyU and Fur, but lacks the direct evidence for their binding to the promoter of *hlyA*. In the present work, we showed that the QS regulator HapR, along with Fur and HlyU, regulates the transcription of *hlyA* in *V. cholerae* El Tor biotype. At the late mid-logarithmic growth phase, HapR binds to the three promoters of *fur, hlyU*, and *hlyA* to repress their transcription. At the early mid-logarithmic growth phase, Fur binds to the promoters of *hlyU* and *hlyA* to repress their transcription; meanwhile, HlyU binds to the promoter of *hlyA* to activate its transcription, but it manifests direct inhibition of its own gene. The highest transcriptional level of *hlyA* occurs at an OD_600_ value of around 0.6–0.7, which may be due to the subtle regulation of HapR, Fur, and HlyU. The complex regulation of HapR, Fur, and HlyU on *hlyA* would be beneficial to the invasion and pathogenesis of *V. cholerae* during the different infection stages.

## Introduction

*Vibrio cholerae*, a Gram-negative and curved bacterium, is the causative agent of the diarrheal disease cholera (Clemens et al., 2017). This pathogen expresses various key virulence factors, including major ones, such as cholera toxin (CT), toxin co-regulated pilus (TCP), flagellum, and cholera hemolysin (HlyA; Almagro-Moreno et al., 2015; Benitez and Silva, 2016; Clemens et al., 2017). HlyA, an extracellular pore-forming toxin, is expressed in the El Tor biotype and most of the non-O1/non-O139 isolates (Yamamoto et al., 1990a; Singh et al., 2001; Diep et al., 2015). It possesses various biological activities including hemolytic activity, lethality, cardiotoxicity, cytotoxicity, and enterotoxicity (Ichinose et al., 1987; Benitez and Silva, 2016). HlyA has been recognized as a virulence determinant in the infant mouse cholera model (Fallarino et al., 2002). Purified HlyA can induce fluid accumulation and a histological change in the mucosa when injected into rabbit ileal loops (Ichinose et al., 1987; Debellis et al., 2009). *In vitro* studies showed that HlyA induces cell vacuolation and apoptosis in cultured mammalian cells (Coelho et al., 2000; Mitra et al., 2000; Figueroa-Arredondo et al., 2001; Chakraborty et al., 2011). HlyA was also strongly suggested to be responsible for lethality, developmental delay, and intestinal vacuoles formation in *Caenorhabditis elegans* during *V. cholerae* infection (Cinar et al., 2010; Sahu et al., 2012).

Expression of HlyA was highly induced when *V. cholerae* was cultured in rabbit ileal loops (Xu et al., 2003). It has also been found to be positively regulated by HlyU, a member of SmtB/ArsR family of transcriptional repressors (Saha and Chakrabarti, 2006). Deletion of *hlyU* decreased HlyA production but increased LD_50_ in the infant mouse cholera model (Williams et al., 1993). HlyU acts as a dimer that binds to the promoter of *hlyA* to activate its transcription (Mukherjee et al., 2015). In addition, the quorum sensing (QS) master regulator HapR was shown to be involved in repressing HlyA expression at both the transcriptional and posttranscriptional levels (Tsou and Zhu, 2010). Repression of *hlyA* by HapR at the transcriptional level was achieved through direct binding of HapR to the *hlyA* promoter, while that at posttranscriptional level was mediated via the metalloprotease HapA (Tsou and Zhu, 2010). However, as a virulence factor, expression of *hlyA* should be under the tight control of multiple regulators.

The ferric uptake regulator Fur is a metal-dependent DNA-binding protein that regulates multiple genes related to metabolism and virulence in *V. cholerae* (Occhino et al., 1998; Davis et al., 2005; Mey et al., 2005; Wyckoff et al., 2007; Davies et al., 2011). A 19 bp palindromic sequence was previously described as the DNA binding box of Fur in *V. cholerae* (Goldberg et al., 1990). However, the palindromic sequence cannot explain all of the DNA-binding characteristics of Fur. Thus, an enhanced *V. cholerae* Fur box with a 21 bp palindromic sequence was constructed based on the ChIP-seq-identified binding sites, but it shares an identical span of bases with the previously predicted (Davies et al., 2011). One Fur box-like sequence, TGAATATCAGTAATTGTTATT, was found within the upstream DNA region of *hlyA*, suggesting that its transcription might be under the direct control of Fur. In the present study, we showed that the highest transcription of *hlyA* occurs at early mid-logarithmic growth phase due to the collective and elaborate regulation of HapR, Fur, and HlyU, suggesting that HlyA would only function during the early mid-logarithmic growth phase in *V. cholerae*. The complex regulatory actions of HapR, Fur, and HlyU on *hlyA* transcription would be beneficial to the invasion and pathogenesis of *V. cholerae*.

## Materials and Methods

### Construction of the Mutants and Complementary Mutants

*Vibrio cholerae* O1 El Tor strain C7258 (Peru, 1991) was used as the wild type (WT) in this study. The deletion mutants of *hapR, fur*, and *hlyU* (designated as *ΔhapR, Δfur*, and *ΔhlyU*, respectively) were constructed from WT using the suicide plasmid pWM91 by allelic exchange, which was similarly performed as previously described (Wu et al., 2015). To construct the complementary mutants, the entire coding region of each deleted gene was cloned into the pBAD24 vector harboring an arabinose PBAD promoter and an ampicillin resistance gene (Guzman et al., 1995; Sun et al., 2014). After being verified by DNA sequencing, the complementary plasmid for each deleted gene was transferred into the corresponding mutant, yielding the complementary mutant strain *ΔhapR*/pBAD24-*hapR, Δfur*/pBAD24-*fur*, or *ΔhlyU*/pBAD24-*hlyU*. In order to counteract the effects of arabinose and ampicillin on bacterial growth, the empty vector pBAD24 was introduced into WT or each mutant to generate WT/pBAD24, *ΔhapR*/pBAD24, *Δfur*/pBAD24, or *ΔhlyU*/pBAD24, respectively (Sun et al., 2014). All the primers used are listed in **Table 1**.

**Table 1:** Oligonucleotide primers used in this study.

Target	Primers (forward/reverse, 5’-3’)
Construction of mutants	
*hapR*	GCGGGATCCCCAGCAATACATCTTTACC/GTGCTGCCCAAGAAAAGGGGTATATCCTTGCC
	GGCAAGGATATACCCCTTTTCTTGGGCAGCAC/GCGACTAGTAACTCACCAAAACCTTC
	GCGGGATCCCCAGCAATACATCTTTACC/GCGACTAGTAACTCACCAAAACCTTC
*fur*	CGGGATCCTTCGTGTAAGGCAGCAGTAATC/CAGAGCGTAAAGCCTATGGATACTTTCCTGTTGATGTTC
	GAACATCAACAGGAAAGTATCCATAGGCTTTACGCTCTG/GGACTAGTAGATGAAGATGGTGTGGGAAAC
	CGGGATCCTTCGTGTAAGGCAGCAGTAATC/GGACTAGTAGATGAAGATGGTGTGGGAAAC
*hlyU*	GCGGGATCCCCAGGCAGTCGAACCGCA/TACCTTTTTTTCGACCACCTTTAATTCCAACCCATTCATTC
	GAATGAATGGGTTGGAATTAAAGGTGGTCGAAAAAAAGGTA/GGACTAGTGAAAGGATAAGAATGTCATAG
	GCGGGATCCCCAGGCAGTCGAACCGCA/GGACTAGTGAAAGGATAAGAATGTCATAG
Construction of complemented mutants	
*hapR*	GATTCTAGAAGGAGGAATTCACCATGGACGCATCAATCGAAAAAC/GCGAAGCTTCTAGTTCTTATAGATACACAG
*fur*	GATTCTAGAAGGAGGAATTCACCATGTCAGACAATAACCAAG/GCGAAGCTTTTATTTCTTCGGCTTGTGAG
*hlyU*	GATTCTAGAAGGAGGAATTCACCATGCCGTATTTAAAGG/GCGAAGCTTCTACTGATTCGCCTGAC
Protein expression	
*hapR*	GCGGGATCCATGGACGCATCAATCGAAAAAC/GCGAAGCTTCTAGTTCTTATAGATACACAG
*fur*	GCGGGATCCATGTCAGACAATAACCAAG/GCGAAGCTTTTATTTCTTCGGCTTGTGAG
*hlyU*	GCGGGATCCATGCCGTATTTAAAGG/GCGAAGCTTCTACTGATTCGCCTGAC
qRT-PCR	
*hapR*	AAACGCAAACTACAACTGATGG/AGCACATCGTCAACCAAGTC
*fur*	AGCCAGAGTGCCAACATATTAG/AATACTGACTTGCCGCCTTC
*hlyU*	CTCAGCCAATCTGCTCTT/AGTTCAATCATCGCCTTC
*hlyA*	CGTTAGATGCCTATTTCCG/CTCCACTGACTTCCACCC
Luminescence assay	
*hapR*	GCGGAGCTCCCAGCAATACATCTTTACC/GCGACTAGTTGAGGCGATAGCCGAGTT
*fur*	GCGGAGCTCGCATCAAGGCATAAACGG/GCGACTAGTATACTTTCCTGTTGATGTTC
*hlyU*	GCGGAGCTCTGTTAGTTCCAGGCAGTC/GCGACTAGTTTTAATTCCAACCCATTC
*hlyA*	GCGGAGCTCCAATCTATGCTTATACGG/GCGACTAGTGCAACGATTGAGTTTTGG
Primer extension	
*hlyU*	/TTTGCAGTCGCCGCTCATTG
*hlyA*	/TCATGGGTTACCCTCGTC
DNase I footprinting	
*fur*	GTAAAACGACGGCCAGTGCATCAAGGCATAAACGG/CAGGAAACAGCTATGACGAGGCAAATCACTGAACAAA
*hlyU*	GTAAAACGACGGCCAGTCGTGTTTATGGCTCCCTC/CAGGAAACAGCTATGACGATGTCGTAATTCGGTTG
*hlyA*	GTAAAACGACGGCCAGTCTTATGTGTAAGCGTATTG/CAGGAAACAGCTATGACCGGATCACAGATTTTAGC
*M13*	(FAM)GTAAAACGACGGCCAGT/(HEX)CAGGAAACAGCTATGAC

### Growth Conditions

The LB broth (1% tryptone, 0.5% yeast extract, and 1% NaCl) was used for *V. cholerae* cultivation. Overnight bacterial cultures were diluted 1:50 into 15 ml of fresh LB broth, and grown under 37°C with shaking at 200 rpm to reach an OD_600_ value of 1.0, and then diluted 1:100 into 15 ml of fresh LB broth for the third-round growth, and were harvested at required cell densities. When necessary, the LB broth was supplemented with 100 μg/ml ampicillin, 5 μg/ml chloramphenicol, 100 μg/ml kanamycin, or 0.1% arabinose.

### Hemolytic Activity Assay

The hemolytic activity of *V. cholerae* strains were tested using the method previously described with slight modifications (Zhang et al., 2017b); 5 μl of the third-round bacterial cultures were transferred onto LB agar containing 5% sheep blood erythrocytes, 100 μg/ml ampicillin, and 0.1% arabinose. The LB blood plates were incubated at 37°C for 20 h.

### Luminescence Assay

For the *lux* activity assay (Xu et al., 2010), the promoter DNA region of each target gene was PCR amplified and cloned into the corresponding restriction endonuclease sites of pBBRlux vector harboring a promoterless *luxCDABE* reporter gene and a chloramphenicol resistance gene. The resulting plasmid was then transferred into WT and mutant strains, respectively. The *V. cholerae* strains transformed with recombinant plasmids were cultivated completely in LB broth at 37°C, and harvested at the required cell densities. The luminescence was measured using an Infinite^®^ 200 Pro NanoQuant (Tecan, Switzerland). The *lux* activity was calculated as light units/OD_600_.

### RNA Isolation and Quantitative Real-Time PCR (qRT-PCR)

Total RNAs were extracted using the TRIzol Reagent (Invitrogen, United States). The cDNAs were generated by using 12 μg of total RNAs and 3 μg of random hexamer primers. The quantitative real-time PCR (qRT-PCR) assay was performed and analyzed as previously described (Gao et al., 2011). The relative mRNA levels were determined based on the standard curve of *recA* (reference gene) expression for each RNA preparation.

### Preparation of 6× His-Tagged Proteins

The entire coding region of *hapR, fur*, and *hlyU* was amplified and cloned into the plasmid pET28a (Novagen, United States), respectively. The resulting plasmids encoding His-tagged proteins were transferred into *Escherichia coli* BL21λDE3 cells for protein expression (Kleber-Janke and Becker, 2000). The methods for purification of His-tagged proteins were done as previously described (Gao et al., 2008; Zhang et al., 2012). The eluted His-tagged proteins were dialyzed and then concentrated to a final concentration of 0.3–0.6 mg/ml. The purity of purified proteins was analyzed by SDS-PAGE.

### DNase I Footprinting

The procedures for DNase I footprinting assay and DNA sequencing were carried out as previously described (Gao et al., 2017; Osei-Adjei et al., 2017; Zhang et al., 2017b). Briefly, upon being incubated with the increasing amounts of His-tagged protein, the FAM (or HEX)-labeled DNA probes were digested by the optimized RQ1 RNase-Free DNase I (Promega). The digested DNA fragments were then analyzed using an ABI 3500XL DNA Genetic analyzer with GeneMarker software 2.2, while the DNA sequencing products were surveyed with Sequence Scanner software v1.0.

### Primer Extension Assay

The primer extension assay was performed as previously described with slight modifications (Gao et al., 2011). Briefly, 12 μg of total RNA was annealed with 1 pmol of 5′-HEX-labeled reverse oligonucleotide primer to generate cDNAs using the Primer Extension System (Promega) according to the manufacturer’s instructions. The primer extension products and sequencing materials were analyzed using the same methods as that of the DNase I footprinting assay.

### Experimental Replicates and Statistical Methods

The presented data of hemolytic activity assay, DNase I footprinting, and primer extension were done at least two independent times. The luminescence assay and qRT-PCR were performed with at least three independent bacterial cultures, and the values were expressed as the mean ± SD. Paired Student’s *t-*test was used to calculate statistically significant differences, and *p* < 0.01 was considered to indicate statistical significance.

## Results

### HapR and Fur Represses the Hemolytic Activity of *Vibrio cholerae*

**FIGURE 1:**
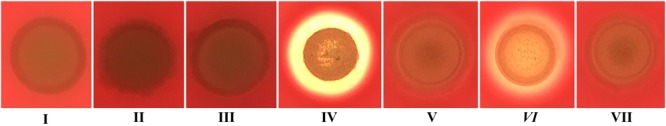
Hemolytic activity of different *Vibrio cholerae* strains on LB-5% sheep blood agar. After being incubated at 37°C for 48 h, the LB blood plates were photographed. I, II, III, IV, V, VI, and VII represent WT/pBAD24, *ΔhlyU*/pBAD24, *ΔhlyU*/pBAD24-*hlyU, ΔhapR*/pBAD24, *ΔhapR*/pBAD24-*hapR, Δfur*/pBAD24, and *Δfur/*pBAD24-*fur*, respectively.

The hemolytic activities against sheep blood erythrocytes were compared between WT/pBAD24, mutant (*ΔhapR*/pBAD24, *Δfur*/pBAD24, or *ΔhlyU*/pBAD24), and the complementary mutant (*ΔhapR*/pBAD24-*hapR, Δfur*/pBAD24-*fur*, or *ΔhlyU*/ pBAD24-*hlyU*) strains (**Figure 1**). The results showed that the WT/pBAD24 and all of the complementary mutants exhibit α-type hemolysis, but *ΔhapR*/pBAD24 and *Δfur*/pBAD24 manifest β-type hemolysis. Thus, both HapR and Fur strongly inhibited the hemolytic activity of *V. cholerae*. Although the *ΔhlyU*/pBAD24 strain also exhibited α-type hemolysis, its colony was much dull than that of WT/pBAD24 or *ΔhlyU*/pBAD24-*hlyU*, which is consistent with HlyU activation of *hlyA* transcription. Taken together, these results indicated that the transcription of *hlyA* would be under the negative control of HapR and Fur, but under the positive regulation of HlyU.

### Transcription of *hapR, fur, hlyU*, and *hlyA* Were All Cell Density-Dependent

**FIGURE 2:**
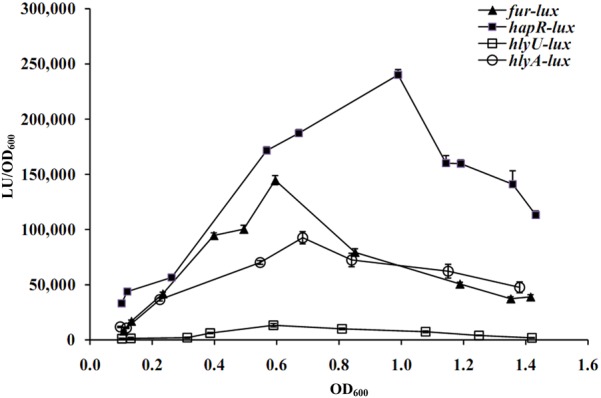
Cell density-dependent expression of target genes. The promoter DNA region of each target gene was cloned into the pBBRlux vector and then transferred into WT to determine the luminescence activity under various OD_600_ values. The bacteria were cultivated completely in LB broth containing the appropriate antibiotics and grown with shaking at 37°C.

The luminescence reporter assay was employed to measure the transcription changes of *hapR, fur, hlyU*, and *hlyA* during the growth periods of the strains (**Figure 2**). The transcription levels of all of the four genes were increased but then reduced with the increase of cell density. In addition, the transcriptional activity of each of the four genes could be detected at all cell densities. However, the highest transcription of *hapR* occurred at an OD_600_ value of around 1.0, while that of *fur, hlyU*, and *hlyA* appeared at an OD_600_ value of around 0.6–0.7. Thus, the *V. cholerae* cells were harvested at the OD_600_ value of about 1.0 and 0.6 for characterizing HapR- and Fur/HlyU-mediated gene regulation, respectively. Moreover, the cell density-dependent transcription of *fur* and *hlyU* suggests that their expression would be under the control of QS.

### HlyU Activates *hlyA* Transcription but Represses Its Own Gene

**FIGURE 3:**
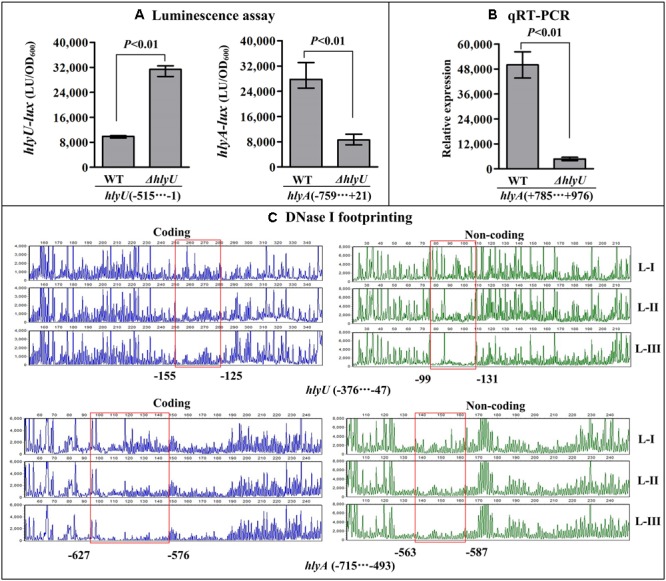
Regulation of *hlyU* and *hlyA* by HlyU. The luminescence assay **(A)** was done as **Figure 2**. **(B)** qRT-PCR. The relative mRNA level of *fur* was compared between *ΔhlyU* and WT. **(C)** DNase I footprinting. The promoter fragment of each target gene was labeled with FAM or HEX, incubated with increasing amounts of purified His-HlyU (Lanes-I, II, and III contain 0, 4.06, and 12.12 pmol, respectively), and then subjected to DNase I footprinting assay. The results were analyzed using an ABI 3500XL DNA analyzer. The protected regions are boxed and marked with positions. The negative and positive numbers indicate the nucleotide positions relative to the translation start site (+1) of each target genes, respectively.

As determined by the luminescence assay (**Figure 3A**), the promoter activity of *hlyA* in *ΔhlyU* was much lower relative to that in WT, whereas that of luminescence under the control of *hlyU* promoter in *ΔhlyU* was much higher than that in WT, suggesting the positive and negative regulation of *hlyA* and *hlyU* by HlyU, respectively. The qRT-PCR assay further confirmed the positive correlation between HlyU and *hlyA* transcription in *V. cholerae* (**Figure 3B**). The DNase I footprinting assay showed that His-HlyU protected a single region, i.e., –563...–627 or –99...–155, for *hlyA* or *hlyU* promoter, respectively, against DNase I digestion in a dose-dependent manner (**Figure 3C**). Taken together, these results suggested that HlyU acts as a transcriptional activator of *hlyA* but serves as a repressor of its own gene in *V. cholerae*.

### Negative Regulatory Actions of HapR and Fur on *hlyU* and *hlyA*

**FIGURE 4:**
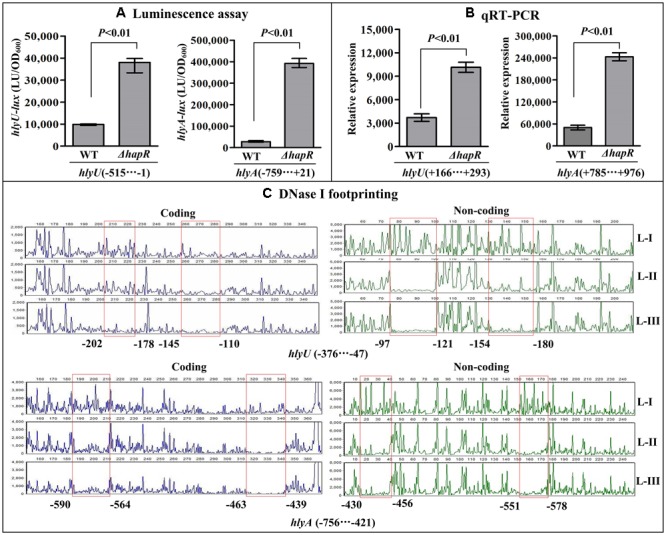
Negatively regulation of *hlyU* and *hlyA* by HapR. The luminescence assay **(A)** was done as **Figure 2**. The qRT-PCR **(B)** and DNase I footprinting assay **(C)** were done as **Figure 3**. Lanes-I, II, and III contain 0, 2.31, and 6.92 pmol His-HapR, respectively.

**FIGURE 5:**
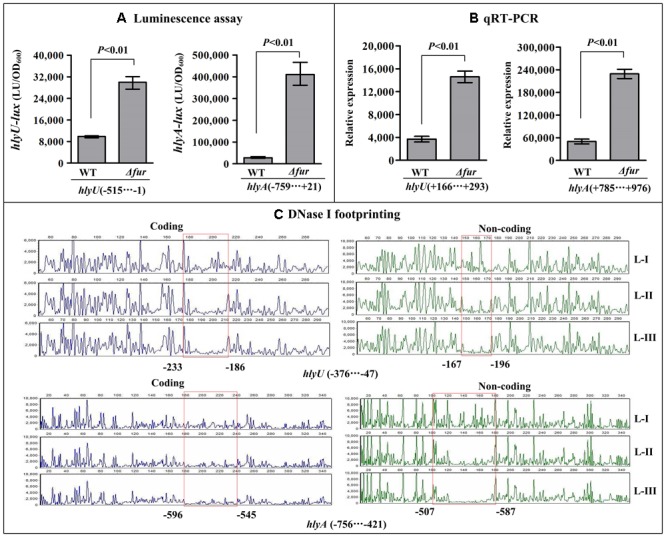
Negatively regulation of *hlyU* and *hlyA* by Fur. The luminescence assay **(A)** was done as **Figure 2**. The qRT-PCR **(B)** and DNase I footprinting assay **(C)** were done as **Figure 3**. Lanes-I, II, and III contain 0, 2.95, and 8.85 pmol His-Fur, respectively.

The results of luminescence assay showed that the promoter activities of *hlyU* and *hlyA* in both *ΔhapR* and *Δfur* were much higher relative to that in WT (**Figures 4A, 5A**). The qRT-PCR assay indicated that the transcriptional levels of *hlyU* and *hlyA* significantly increased in *ΔhapR* and *Δfur* relative to WT (**Figures 4B, 5B**). The DNase I footprinting assay disclosed that His-HapR protected two different DNA regions for each promoter against DNase I digestion (**Figure 4C**), while His-Fur only protected a single region for each promoter (**Figure 5C**). Taken together, both HapR and Fur repressed the transcription of *hlyU* and *hlyA* in a direct manner.

### Identification of the Transcription Start Sites for *hlyA* and *hlyU*

**FIGURE 6:**
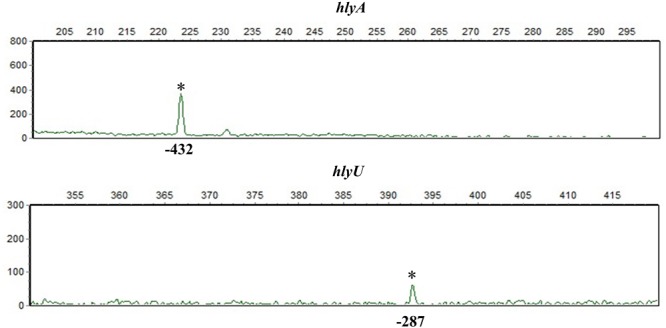
Transcription start sites of *hlyA* and *hlyU*. A 5’-HEX-labeled oligonucleotide primer was designed to be complementary to the RNA transcript of each target gene. The primer extension products were analyzed with an ABI 3500XL DNA Genetic analyzer. The transcription start sites are marked with asterisks and positions.

Two transcriptions start sites of *fur* have been previously reported in *V. cholerae* (also seen in **Figure 8**; Litwin et al., 1992; Lam et al., 1994). In the present work, the primer extension assay was employed to map the transcription start sites of *hlyA* and *hlyU*. The assay detected only one transcription start site for each gene located at 287 bp upstream of *hlyA* and 432 bp upstream of *hlyU*, respectively (**Figure 6**).

### HapR Represses *fur* Transcription

**FIGURE 7:**
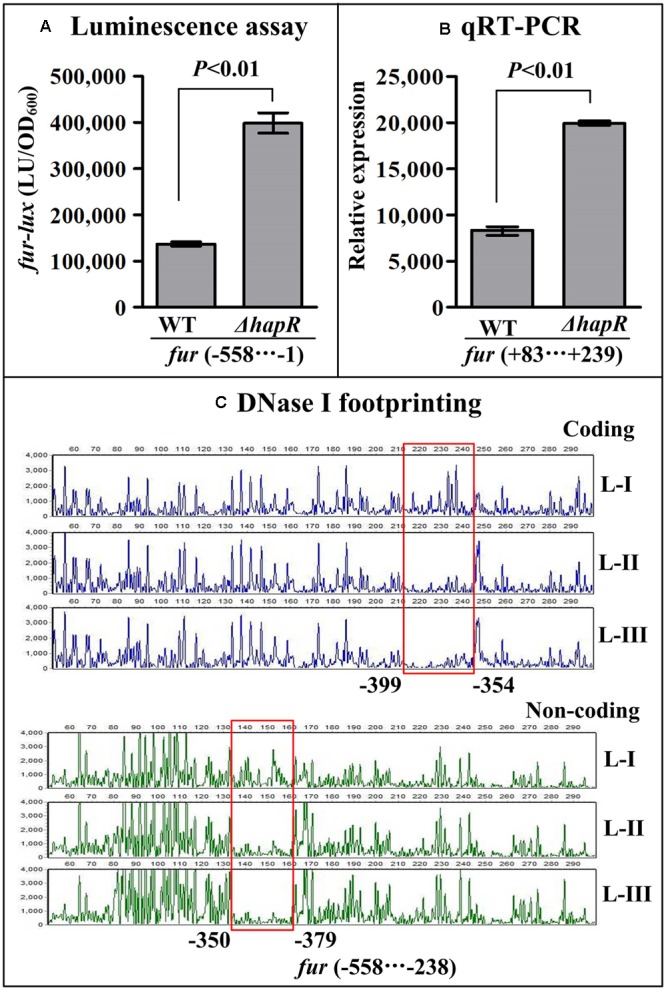
Transcription of *fur* was negatively regulated by HapR. The luminescence assay **(A)** was done as **Figure 2**. The qRT-PCR **(B)** and DNase I footprinting assay **(C)** were done as **Figure 3**.

The recombinant pBBRlux plasmid that contains the promoter-proximal region of *fur* and a promoterless *luxCDABE* reporter gene was transferred into *ΔhapR* and WT, respectively, to test the action of HapR on the promoter activity of *fur*. As shown in **Figure 7A**, the promoter activity of *fur* in *ΔhapR* was much higher relative to that in WT, indicating the negative correlation of HapR and *fur* transcription in *V. cholerae*. As further determined by the qRT-PCR assay (**Figure 7B**), the mRNA level of *fur* was significantly enhanced in *ΔhapR* relative to WT. The results of *in vitro* DNase I footprinting (**Figure 7C**) demonstrated that His-HapR protected a single region from 238 to 558 bp upstream of *fur* against DNase I digestion in a dose-dependent manner. Taken together, HapR directly and negatively regulates the transcription of *fur* in *V. cholerae*.

## Discussion

**FIGURE 8:**
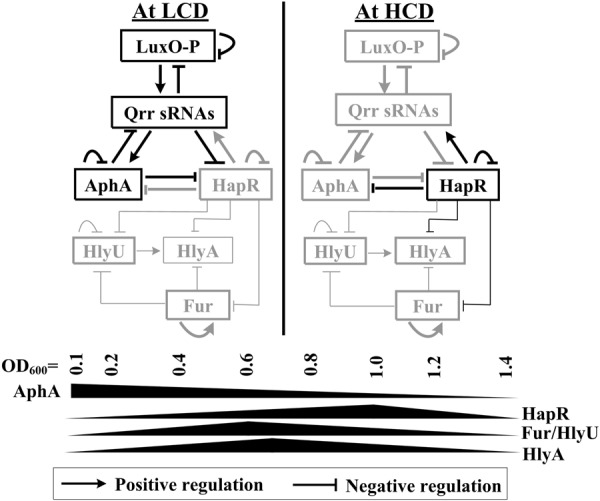
Regulatory circuit. The regulatory actions between LuxO, Qrr sRNAs, AphA, and HapR were previously described in *Vibrio cholerae* and closely related vibrios (Henke and Bassler, 2004; Tu et al., 2010; Rutherford et al., 2011; Sun et al., 2012; Zhang et al., 2012). AphA and HapR are the two master QS regulators operating at LCD and HCD, respectively. Fur and HlyU, which are transcribed highly at an OD_600_ value of around 0.6, coordinate with HapR to tightly regulate *hlyA* transcription, leading to the high expression of HlyA at the early mid-logarithmic growth phase. Positive autoregulation of Fur has been established in *V. vulnificus* (Lee et al., 2007); this mechanism would be conserved between *V. vulnificus* and *Vibrio cholerae*.

Although iron-, Fur-, HapR-, and HlyU-dependent expression of *hlyA* has been previously reported in *V. cholerae* (Stoebner and Payne, 1988; Williams et al., 1993; Tsou and Zhu, 2010; Mukherjee et al., 2015), the detailed regulatory mechanisms need to be further illustrated. In this study, we showed that the transcription of *hlyA* was regulated coordinately by HlyU, HapR, and Fur in *V. cholerae* El Tor biotype (**Figure 8**). At the late mid-logarithmic growth phase (OD_600_ ≈ 1.0), the highly expressed HapR bound to the promoters of *fur, hlyU*, and *hlyA* to repress their transcription. At the early mid-logarithmic growth phase (OD_600_ ≈ 0.6), the highly expressed Fur bounds to the promoters of *hlyU* and *hlyA* to repress their transcription; meanwhile, HlyU bounds to the promoters of *hlyA* and *hlyU* to activate and inhibit their transcription, respectively. The highest transcriptional level of *hlyA* occurred at an OD_600_ value of about 0.7 due to the tight regulation of HapR, Fur, and HlyU, suggesting that HlyA would function at the early mid-logarithmic growth phase in *V. cholerae*.

*Vibrio vulnificus* secretes a potent hemolysin (VvhA) sharing homologous regions with HlyA (Yamamoto et al., 1990b). VvhA exhibits strongly cytolytic and hemolytic activities and may contribute to the bacterial invasion and causes vasodilatation (Kim et al., 1993; Kook et al., 1996; Elgaml and Miyoshi, 2017). *Vibrio vulnificus* SmcR, a HapR homolog, directly represses the expression of *hlyU* and *vvhA*, while HlyU directly activates *vvhA* transcription (Shao et al., 2011; Wen et al., 2012). Deletion of *hlyU* resulted in the loss of cytotoxicity and reduced VvhA production in the *smcR* mutant (Shao et al., 2011). The double mutant of *smcR* and *hlyU* regained cytotoxicity and hemolytic activity when *hns* was further deleted (Shao et al., 2011). HlyU seems act as an anti-repressor of H-NS in the regulation of the virulence genes in *V. vulnificus* (Liu et al., 2009). In addition, it has been shown that iron represses *vvhA* transcription via Fur, which represses *vvsA* transcription in the presence of iron through the protein–promoter DNA association (Kim et al., 2009). The binding site of Fur overlaps with that of SmcR but with a higher affinity than SmcR (Wen et al., 2012). Moreover, Fur has been shown to be involved in the regulation of *smcR* transcription in *V. vulnificus* (Kim et al., 2013; Wen et al., 2016). However, *V. cholerae* Fur seems to have no regulatory activity on *hapR* transcription (data not shown), suggesting that the regulation of the QS regulator gene by Fur may depend on the bacterial growth conditions. Nevertheless, the conservative regulatory mechanisms might be employed to tightly control of the HlyA production in *V. vulnificus* and *V. cholerae*.

**FIGURE 9:**
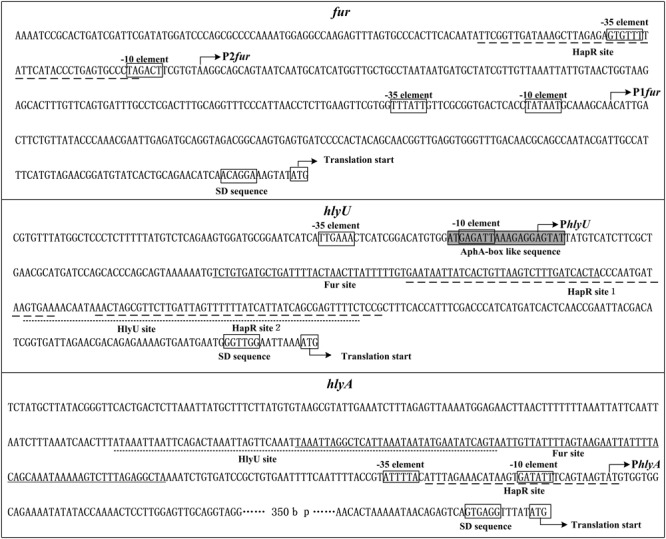
Structural organization of target promoters. The DNA sequence was derived from *Vibrio cholerae* El Tor C7258. The transcription/translation start sites are indicated by bent arrows. Shine-Dalgarno (SD) box and –10/–35 elements are enclosed in boxes. The HapR sites are underlined with broken lines, the Fur sites are underlined with solid lines, while the HlyU sites are underlined with dotted lines.

The transcription of *hlyA, fur*, and *hlyU* was stimulated at the early mid-logarithmic growth phase but repressed at both low cell density (LCD) and high cell density (HCD; **Figure 2**), suggesting that some unknown regulators can repress their transcription at LCD. AphA has been considered as the bottom master regulator of QS operating at LCD (Ng and Bassler, 2009; Ball et al., 2017). The DNA binding box of AphA has been identified as an inverted repeat of ATATGC with a 6-nt centered spacer, i.e., ATATGCA-N6-TGCATAT (Sun et al., 2012). An AphA box-like sequence (ATACTCCTCTTTAATCTCAT) was detected within the promoter of *hlyU* but was not found in the other two promoters (**Figure 9**). Thus, the transcription of *hlyU* would be under the direct control of AphA. The asymmetric production of AphA and HapR orthologs coupled with their combined inhibition of downstream targets has been observed in other vibrios (Van Kessel et al., 2013; Zhang et al., 2017a). For example, in *Vibrio harveyi*, both AphA and LuxR bound to the promoters of the type III secretion system (T3SS) genes to repress their transcription, resulting in the highest expression levels of T3SS occurring at LCD-to-HCD transition (Van Kessel et al., 2013); in *Vibrio parahaemolyticus*, ToxR coordinates with AphA and OpaR to repress T6SS1, leading to the highest expression of T6SS1 occurring at the mid-logarithmic growth phase (Zhang et al., 2017a). However, the detailed regulatory mechanisms of AphA or other additional factors on *hlyA* transcription in *V. cholerae* need to be further investigated.

The organization of *fur, hlyU*, and *hlyA* promoters was reconstructed herein, by collecting the data of translation/transcription start sites, promoter -10 and -35 elements, HapR/Fur/HlyU binding sites, AphA box-like sequence, and Shine-Dalgarno (SD) sequences (ribosomal binding sites; **Figure 9**). One HapR binding site was detected in the upstream of *fur* and *hlyA*, respectively, and each overlaps the core -10 element; two HapR binding sites for *hlyU* were detected and both of them were located downstream of the transcription start site. Thus, the binding of HapR would block the entry or elongation of the RNA polymerase to repress the transcription of the target genes. Both the Fur and HlyU sites for *hlyU* are located downstream of the transcription start site, indicating that the repression mechanisms of *hlyU* by Fur and HlyU would be similar to that by HapR. Notably, the Fur site for *hlyU* overlaps the HapR site 1, while HlyU site overlaps the HapR site 2. Thus, there may be a competitive binding activity between HapR and Fur or HlyA in the binding of the *hlyU* promoter. Although the binding site of Fur to *hlyA* is located upstream of the transcription start site, it overlaps with that of HlyU. Thus, the binding of HapR would block the binding of HlyU, which acts as a transcriptional activator of *hlyA* in *V. cholerae*.

## Conclusion

This work reports that QS coordinates with HlyU and Fur to regulate *hlyA* transcription in *V. cholerae*, leading to the highest transcription of *hlyA* occurring at the early mid-logarithmic growth phase when bacteria cells are grown in LB broth. Therefore, we propose that at the early or middle stage of infection, *V. cholerae* produces high amount of HlyA in the small intestine, which promotes the bacterial invasion and pathogenesis, and contributes to the watery diarrhea; at the end of the infectious cycle, since HapR is highly expressed, it activates the protease production and inhibits the biofilm formation, to detach the mutual aggregation of *V. cholerae* cells in the initial infection sites in the intestine. Meanwhile, the expression of CT decreases, and therefore the shift to lower expression of HlyA may have a similar response of CT.

## Author Contributions

HG, JX, YZ, and BK conceived the study and designed experimental procedures. XL, JLi, JLo, HZ, BD, and QS performed the experiments and carried out data analysis. HG, YZ, and BK wrote the paper.

## Conflict of Interest Statement

The authors declare that the research was conducted in the absence of any commercial or financial relationships that could be construed as a potential conflict of interest. The reviewer RW and handling Editor declared their shared affiliation.
